# Obese Women Have a High Carbohydrate Intake without Changes in the Resting Metabolic Rate in the Luteal Phase

**DOI:** 10.3390/nu14101997

**Published:** 2022-05-10

**Authors:** Eduard Maury-Sintjago, Alejandra Rodríguez-Fernández, Julio Parra-Flores, Marcela Ruíz-De la Fuente

**Affiliations:** 1 Department of Nutrition and Public Health, Universidad del Bío-Bío, Chillan 3780000, Chile; emaury@ubiobio.cl (E.M.-S.); alrodriguez@ubiobio.cl (A.R.-F.); juparra@ubiobio.cl (J.P.-F.); 2 GABO Grupo de Investigación en Auxología, Bioantropología y Ontogenia, FACSA, Universidad del Bío-Bío, Chillan 3780000, Chile

**Keywords:** resting metabolic rate, dietary intake, menstrual cycle, nutritional status

## Abstract

Hormonal changes are caused by the menstrual cycle phases, which influence resting metabolic rate and eating behavior. The aim of the study was to determine resting metabolic rate (RMR) and its association with dietary intake according to the menstrual cycle phase in lean and obese Chilean women. This cross-sectional analytical study included 30 adult women (15 lean and 15 with obesity). Body composition was measured with a tetrapolar bioelectrical impedance meter. Nutritional status was determined by adiposity. A 24-h recall of three nonconsecutive days verifies dietary intake. The RMR was measured by indirect calorimetry. All measurements were performed in both the follicular and luteal phases of the menstrual cycle. Statistical analyses were performed with STATA software at a significance level, which was α = 0.05. The RMR (β = 121.6 kcal/d), temperature (β = 0.36 °C), calorie intake (β = 317.1 kcal/d), and intake of lipids (β = 13.8 g/d) were associated with the luteal phase in lean women. Only extracellular water (β = 1.11%) and carbohydrate consumption (β = 45.2 g/d) were associated in women with obesity. Lean women showed increased RMR, caloric intake, and lipid intake during the luteal phase. For women with obesity, carbohydrate intake increased but not RMR.

## 1. Introduction

Obesity is a pathological condition that has been steadily increasing over several decades in most westernized countries in which 60–80% of adults are overweight [1]. Prevalence has increased in both adults and children, regardless of geography, locality, ethnic group, or socioeconomic level; however, a higher prevalence has been observed in women living in low-income countries [2].

Despite the multifactorial nature of obesity, it influences genetic and nutritional aspects and lack of physical activity [3]; however, the energy imbalance has been described as one of the main causes due to the dynamic interrelationship between various components of energy balance and compensatory physiological processes [4]. The resting metabolic rate (RMR) contributes as much as 60–75% of the total daily energy expenditure, and it plays a key role in energy balance and body weight control [5]. The resting energy expenditure (REE) is strongly conditioned by both fat-free mass and body fat; therefore, REE in subjects with obesity increases more than REE in lean subjects [6].

The RMR in women is affected by the menstrual cycle [7,8,9]. It has been directly associated with the secretion of sex hormones during the menstrual cycle, and these influences both energy metabolism and appetite and weight control [10,11]. However, contradory findings have been reported with lower RMR during the follicular phase and higher in the luteal phase [12] or no difference [13]. In their systematic review, Benton et al. [14] reported that much of the research has been restricted to the study of the follicular phase, with design biases related to the control of the menstrual cycle; furthermore, women with obesity were not included

In addition to RMR changes, dietary and caloric intake can also differ during the menstrual cycle [11]. Birgisdóttir et al. [15] showed an excessive intake of sugars and fiber during the menstrual cycle. These findings concur with those reported by Bronzi et al. [16], in a study of Brazilian women, who showed an increased intake of sugars, fats, and salt during the luteal phase. However, there is still no consensus on the impact of the menstrual cycle on caloric or nutrient intake because some studies show no differences [17,18].

To date, there are no publications on RMR during the menstrual cycle that study women with obesity in Latin America. Therefore, the objective of the present study was to determine the association of resting metabolic rate with dietary intake during the menstrual cycle in lean and obese Chilean women.

## 2. Materials and Methods

### 2.1. Study Design

This was an analytical and cross-sectional study.

### 2.2. Sample

The sample consisted of 30 adult women aged 18–25 years, 15 with obesity and 15 lean. All the participants had a regular menstrual cycle and the menstrual cycle phase was verified by reviewing the medical record. The following exclusion criteria were applied to select the sample: irregular menstrual cycle, body weight variation greater than 10% in the last 3 months, use of weight control medication, suspected pregnancy, and breastfeeding. All the women had to be in apparent good health, so that those with hormonal or hypermetabolic pathologies or anemia were excluded, as were those who consumed drugs or medications that modified resting energy expenditure (REE).

The study was reviewed and approved by the Ethics and Biosafety Committee of the University of the Bío-Bío (Chile). All participants gave informed consent. The procedures were in accordance with the ethical standards of the Declaration of Helsinki and the Council of International Organizations of Medical Sciences (CIOMS) [19].

### 2.3. Nutritional Status

Body weight was measured first thing in the morning after urinating during the follicular phase between days 6 and 13 and during the luteal phase between days 15 and 28. A previously calibrated electronic scale (SECA, model 876) with a 100 g graduation and ±0.5% accuracy was used, and it was located in a fixed place on a flat surface. The participants at the time wore no shoes and only underwear, and they stood on the center of the scale, upright with the shoulders back, arms hanging loosely at their sides without exerting pressure, heels together, and toes apart.

Body composition (percentage of fat mass) was determined by bioelectrical impedance (Bodystat Quadscan 4000) [20]. The percentage fat mass classified the participants as lean (20–30%) and with obesity (>30%) [21]. In addition, fat-free mass (% and kg), fat mass (% and kg), total water (% and L), intracellular water (% and L), and extracellular water (% and L) measurements were recorded.

### 2.4. Dietary Intake

All the participants were evaluated for caloric and nutritional intake by applying a 24-h recall survey of 3 nonconsecutive days, which included 2 days of weekday and 1 weekend day. Caloric and nutritional intake were measured at two time points: the follicular phase (days 6–13) and luteal phase (day 15–28) of the menstrual cycle [14].

### 2.5. Indirect Calorimetry

The RMR was measured by indirect calorimetry (IC) with a Vmax 29n (SensorMedics Corp., Yorba Linda, CA, USA), which was calibrated before each measurement. The environmental CO_2_ concentration was maintained at less than 3%, and the room temperature at 20–24 °C. Participants were asked not to consume caffeine smoke or exercise starting the day before the IC measurement. At the time of the IC measurement, fasting was verified (10–12 h), participants rested for 30 min, and vital signs were controlled: axillary body temperature less than 37.0 °C and respiratory rate at 12–18 breaths per minute. The Vmax 29n canopy was then placed on the participant´s head; the expired air was measured until a stable state was achieved, and the validation requirements were met [22,23].

The reading included the volume of CO_2_ production (VCO_2_), the volume of O_2_ consumption (VO_2_), and the respiratory quotient (RQ), which is the CO_2_ production: O_2_ consumption ratio. The IC test was validated by the RQ, which was expected to show a normal physiological range of 0.7–1.0 [22], and by checking CO_2_ exchange (mL/min) and O_2_ (mL/min) exchange fluctuations [24]. The IC was performed during baseline measurements with a metabolic cart for 25 min, including a 5-min stabilization period at the beginning. The steady state was defined as the first 5-min period with a coefficient of variation (CV) ≤ 10% for both VO_2_ and VCO_2_ [25].

### 2.6. Statistical Analysis

The information was analyzed with descriptive and inferential statistics. Percentiles were used to compare the anthropometric and body composition variables related to the menstrual cycle phase using the Wilcoxon or Mann–Whitney test for lean and obese women. An ANOVA model with linear fit was generated using the general linear model (GLM) for each variable under study and for both lean and obese women. The linear coefficient associated with each response variable, standard error, and significance of the variable in the model was calculated according to the *t*-test. Homogeneity of variance was determined according to the Levene test. The analysis was performed in STATA 16.0 software. All tests had a significance level of α = 0.05.

## 3. Results

Table 1 shows that neither group showed differences in body weight and body composition between the follicular and luteal phases of the menstrual cycle (*p* > 0.05). Lean women showed a higher intake of calories and lipids and a higher body temperature in the luteal phase than in the follicular phase (*p* < 0.05). Meanwhile, women with obesity showed a significantly higher intake of calories and carbohydrates in the luteal phase than in the follicular phase (*p* < 0.05). The RMR was higher in the luteal phase only in lean women (*p* = 0.004).

Table 2 shows that the luteal phase is with higher RMR (β = 121.6 kcal/d), body temperature (β = 0.36 °C), caloric intake (β = 317 kcal/d), and lipid intake (β = 13.8 g/d) in lean women (*p* < 0.05). For women with obesity, a positive association was found only during the luteal phase with the increase in the percentage of extracellular water (β = 1.11%) and carbohydrate intake (β = 45.2 g/d) (*p* < 0.05).

## 4. Discussion

Women experience more difficulties than men to control body weight due to the sexual dimorphism of body composition [26]. During the menstrual cycle, the secretion of sex hormones directly influences energy metabolism [10] and appetite control [11].

In the present study, the group of lean women showed an increase in RMR in the luteal phase compared with the follicular phase (*p* < 0.05), while women with obesity showed no change. Increased RMR in the luteal phase has also been reported in other studies and has been mainly associated with higher estrogen level [10,14,26,27,28]. It has been indicated that women with obesity have lower progesterone and estrogens levels [29]. Estrogen is related to the modulation of eating behavior, enhanced expression of uncoupling protein-1 (UCP-1), increased energy expenditure, and lower cardiometabolic risk [30,31].

Our findings study revealed that only lean women experienced body temperature in the luteal phase. It has been reported that body temperature in this phase is conditioned by the progesterone level, which is lower in women with obesity [32]. Likewise, a higher body temperature could be related to a higher RMR, given that each additional degree of body temperature above 35.5 °C, the RMR increases by 7% [33].

The analysis of body composition showed no differences between phases in both groups: this result is similar to previously published findings [34]. However, in women with obesity, the percentage of extracellular water increased during the luteal phase, which is consistent with the anabolic function of estrogen that leads to sodium and water retention [35].

Our study showed increased caloric and lipid intake in lean women during the luteal phase compared with women with obesity, who only increased carbohydrate intake. Few studies have analyzed nutrient intake according to adiposity in the luteal and follicular phases, although higher caloric intake [15,18,36,37] and intake of sweet foods have been reported in the luteal phase [16,38]. However, the available literature has shown contradictory results [27].

It is important to highlight that increased caloric and lipid intake in lean women occurs simultaneously with increased RMR, which could help this group maintain body weight [39]. In contrast, women with obesity do not increase their RMR in the luteal phase, which could lead to increased body weight and metabolic deterioration due to a higher glycemic load in a phase where a lower sensitivity to insulin has also been reported [40,41,42]. Excessive sugar consumption has been associated with diabetes and cognitive impairment, which are mediated by an increase in the inflammatory process [43].

Given the global epidemiological profile and the high obesity rates, it is necessary to further investigate the interrelationship between body composition, energy expenditure, and menstrual cycle. The present study incorporated methodological and design aspects that have not been considered in previous studies, such as regular menstrual cycles, adiposity, and measurement instruments.

Our study has some limitations that should be considered to correctly interpret the results, including the fact that we did not measure sex hormones, incorporated only one menstrual cycle, and studied women with obesity who had body fat percentages no higher than approximately 30%.

Our results suggest that, from a clinical applicability perspective, it is necessary to consider body composition in the nutritional diagnosis instead of the body mass index (BMI), which is a low-sensitivity indicator for determining obesity [44]. It also seems to be more convenient for women with obesity to begin a diet to control body weight during the follicular phase rather than the luteal phase. The caloric intake and consumption of carbohydrate-rich foods are lower in the follicular phase, and this promotes adherence to the diet. 

## 5. Conclusions

Lean women had a higher resting metabolic rate (RMR), energy intake, and lipid intake during the luteal phase. Meanwhile, women with obesity increased their carbohydrate intake but not their RMR; this could lead to a positive energy balance and make it difficult to control body weight over time.

## Figures and Tables

**Table 1:** Weight, rest metabolic rate, body composition, and nutritional intake of lean and obese women according to the menstrual cycle phase.

Nutritional Status	Variables	Follicular Phase			Luteal Phase			*p*
		P25	P50	P75	P25	P50	P75	
Lean	Weight (kg)	54.0	56.4	57.9	54.7	56.3	58.9	0.990
	RMR (Kcal/day)	987	1080	1095	1102	1199	1319	0.004 *
	FFM (%)	71.8	73.5	74.1	71.6	73.2	75.1	0.983
	FFM (kg)	40.0	41.7	42.9	39.9	41.8	42.9	0.950
	FM (%)	25.9	26.5	28.2	24.9	26.8	28.4	0.984
	FM (kg)	14.1	15.3	16.1	13.4	15.7	16.2	0.618
	Total Water (%)	50.9	51.6	52.9	50.9	51.7	53.7	0.693
	Total Water (Lt)	27.7	29.2	30.4	28.8	29.0	30.2	0.802
	Intracellular Water (%)	27.0	27.3	28.0	26.9	27.1	27.4	0.244
	Intracellular Water (Lt)	14.6	15.5	16.4	14.4	15.2	16.2	0.519
	Extracellular Water (%)	23.5	24.2	25.1	23.6	24.0	26.1	0.518
	Extracellular Water (Lt)	13.1	13.6	14.2	13.4	13.8	14.4	0.406
	Temperature (°C)	36	36.1	36.3	36.1	36.6	36.7	0.015 *
	Respiratory quotient	0.8	0.94	1.03	0.76	0.89	0.95	0.253
	Calories (Kcal/day)	1472	1764	1978	1760	2003	2310	0.040 *
	Proteins (g./day)	52.3	63.4	88.5	57.8	72.4	87.0	0.633
	Carbohydrates (g./day)	198.8	246.1	289.5	213.3	239.6	290.8	0.443
	Lipids (g./day)	50.7	64.4	78	62.7	73.0	83.0	0.044 *
Obese	Weight (kg)	61.5	66.2	69.2	62.7	67.5	69.0	0.787
	RMR (Kcal/day)	1115	1221	1308	1148	1244	1312	0.520
	FFM (%)	67.3	68.9	69.3	55.8	68.3	70.4	0.967
	FFM (kg)	42.6	43.8	47.5	42.7	44.2	47.9	0.708
	FM (%)	30.7	31.1	32.7	29.6	31.7	33.2	0.976
	FM (kg)	18.9	20.8	21.5	19.3	20.1	22.1	0.977
	Total water (%)	46.5	47.1	48.2	47.5	47.8	48.4	0.253
	Total water (Lt)	29.4	30.6	32.2	29.2	31.1	32.6	0.663
	Intracellular water (%)	25.0	25.7	26.0	23.2	25.9	26.2	0.917
	Intracellular water (Lt)	16.2	17.2	17.4	15.3	17.1	17.9	0.983
	Extracellular water (%)	21.9	22.3	22.6	22.4	22.7	23.2	0.149
	Extracellular water (Lt)	13.9	14.6	15.4	14.3	15.2	16.4	0.157
	Temperature (°C)	35.4	36	36.2	35.9	36.2	36.4	0.129
	Respiratory quotient	0.73	0.81	0.9	0.75	0.79	0.83	0.868
	Calories (Kcal/day)	1504	1684	1955	1751	1952	2248	0.036
	Proteins (g/d)	53.8	60.8	76.0	61.3	63.3	80.2	0.272
	Carbohydrates (g/d)	192.7	213.3	229.0	222.7	238.4	304.0	0.004 *
	Lipids (g/d)	53.4	63.2	76.0	66.7	72.0	82.3	0.065

* Significant at *p* < 0.05. RMR: Resting metabolic rate; FFM = fat-free mass; FM = fat mass; P 25: 25th percentile; P 50: 50th percentile; P 75: 75th percentile.

**Table 2:** Beta coefficient (β) estimated from the fit of an ANOVA model using a general linear model for each variable in the luteal phase compared with the follicular phase in lean and obese women.

Variables	Lean			Obese		
	Coefficient (β)	SE	*p*	Coefficient (β)	SE	*p*
Weight (kg)	−0.03	1.54	0.986	0.43	2.6	0.870
RMR (kcal/d)	121.6	38.8	0.004	29.5	50.9	0.568
FM (%)	−0.08	0.74	0.908	−0.08	1.39	0.954
FM (kg)	0.41	0.58	0.484	0.08	1.77	0.965
FFM (%)	0.08	0.74	0.908	0.61	1.57	0.702
FFM (kg)	−0.05	1.25	0.968	0.36	1.35	0.794
Total water (%)	0.52	0.79	0.520	0.41	0.95	0.675
AIC (%)	−1.33	1.09	0.232	−0.60	0.58	0.302
AIC (L)	−0.71	0.74	0.344	0.17	0.84	0.838
ECW (%)	1.48	1.21	0.231	1.11	0.52	0.041
ECW (L)	0.85	0.71	0.236	0.7	0.45	0.135
Temperature (°C)	0.36	0.14	0.013	0.28	0.16	0.094
RQ	−0.05	0.43	0.278	−0.003	0.03	0.939
Calories (kcal/d)	317.1	149.2	0.043	270	138.0	0.061
Proteins (g/d)	3.38	9.9	0.735	3.65	8.16	0.658
Carbohydrates (g/d)	17.4	19.2	0.372	45.2	19.0	0.025
Lipids (g/d)	13.8	6.01	0.029	8.07	7.6	0.297

RMR = resting metabolic rate; FM = fat mass; FFM = fat-free mass; AIC = intracellular water, ECW = extracellular water; RQ = respiratory quotient.

## Data Availability

The data presented in this study are available on request from the corresponding author.
